# Detection of Lunar Regolith Acquired by Excavator Using Radiofrequency (RF) Sensors

**DOI:** 10.3390/s25030751

**Published:** 2025-01-26

**Authors:** Krzysztof Kurek, Karol Seweryn, Arkadiusz Tkacz, Gunter Just

**Affiliations:** 1 Institute of Radioelectronics and Multimedia Technology, Warsaw University of Technology, 00-665 Warsaw, Poland; krzysztof.kurek@pw.edu.pl; 2 Space Research Centre Polish Academy of Sciences, 00-716 Warsaw, Poland; kseweryn@cbk.waw.pl; 3 Robotics Section, ESTEC, European Space Agency (ESA), 2201 AZ Noordwijk, The Netherlands

**Keywords:** Moon exploration, RF sensors, regolith (soil) mass determination, regolith sampling/acquisition

## Abstract

This paper presents the concept of a radiofrequency (RF) sensor designed to estimate the mass of the regolith acquired by a sampling device or excavator in planetary environments. The sensor utilizes a microstrip line with an open end as the sensing element, with the mass estimation based on measurements of the phase of the reflection coefficient (S11 of the scattering matrix) for the line immersed in the regolith. The Rotary Clamshell Excavator (RCE) was employed for the experimental evaluation of the sensor’s performance. The RCE successfully passed an environmental test campaign, demonstrating its suitability for future lunar missions. The test results indicate that the RF sensor can estimate the mass of the acquired regolith with reasonable accuracy, approximately 15%, making it a viable solution for rough mass estimation in sampling devices and excavators.

## 1. Introduction

Preparations for a permanent human presence on the Moon are becoming more realistic and will occur within the next decade. Key space agencies have their own plans, such as the Artemis missions [1] established by NASA, the Terrae Novae 2030 strategy developed by the European Space Agency (ESA) [2], and the Chinese Chang’e program [3]. The establishment of a Moon settlement will require a significant civil engineering and mining endeavor [4]. For the initial phase, only a limited number of astronauts will be present on the Moon [5], resulting in a greater dependence on automated construction techniques compared to terrestrial conditions. A number of solutions for the automated or autonomous construction of bases have been proposed in recent years, including technologies such as (i) 3-D printing of concrete-like composites [6], (ii) geopolymer-like composites [7], (iii) inflatable structures covered by the regolith available on the Moon [8], and (iv) the utilization of natural caves [9]. For most of these techniques, a significant amount of regolith is needed and therefore there is a strong need to learn how to excavate, handle, and transport lunar regolith.

The Moon’s environment (see the details in [10]) poses a number of significant challenges that need to be overcome during lunar settlement development. The Moon’s exosphere is at an ultra-low pressure (3 × 10^−13^ Pa—practically a vacuum) with levitated electromagnetic dust, experiences one-sixth of terrestrial gravity, and undergoes extreme temperature fluctuations caused primarily by the day/night cycle ranging from approximately −170 °C to +130 °C. The lunar landscape is strewn with impact craters, volcanoes, lava flows, lava-filled depressions, hills, and numerous rock faults. Continuous exposure to meteorites, comets, and micrometeorite bombardment for billions of years has resulted in a regolith layer of varying thickness, ranging from 4 to 5 m in the lunar mare regions and from 10 to 12 m in the lunar highlands [11]. The composition of the regolith and its geotechnical parameters are relatively well known thanks to samples brought back by the LUNA, Apollo and Chang’e missions. The main components are as follows: SiO_2_—45.4%, Al_2_O_3_—14.9%, FeO—14.1% and CaO—11.8% [12]. There are three important parameters impacting the geotechnical properties of the regolith: (i) a practically linear particle size distribution ranging from single µm up to mm [13], (ii) a relative density ranging from 65% in the first few cm below the surface up to 90% below 30 cm and a bulk density that can be up to 1.9 g/cm^3^, and (iii) the highly angular shapes of the grains found in agglutinates and non-molten particles generated during meteorite impacts.

During the LUNA and Apollo lunar programs, core drilling was carried out with the use of specially designed devices. The deepest boreholes to date have been carried out by the crewed Apollo 15, 16, and 17 missions on the Moon. The boreholes were drilled using the Apollo Lunar Surface Drill (ALSD) percussion mechanism to a maximum depth of 3 m, and the cores were taken from the entire length of the hole. The regolith was also delivered to Earth by the Soviet missions LUNA 16, 20, and 24, which provided about 300 g of material from the Moon [14]. In recent years, China realized several successful robotic missions to the lunar surface. The Chang’e 4 mission brought about a new insight into the subsurface structure of the Moon. Thanks to the Lunar Penetrating Radar (LPR) device installed on board, it was possible to understand the stratigraphic structure of the rock layers in the Von Karman Crater down to a depth of 40 m [15]. In 2020, the Chang’e 5 mission delivered 1.731 g of lunar regolith to Earth, and recently [16], the Chang’e 6 mission delivered almost 2 kg of lunar regolith from the far side region to Earth.

The space missions listed above are the final goal of technology development conducted in terrestrial or laboratory conditions. In the context of geotechnical activities after the Apollo missions to the Moon, different regolith simulants (also called regolith analogues) have been developed for terrestrial conditions. A summary of this effort was given by Taylor et al. (2016) [17]. In parallel, there exists a database of lunar simulant prepared by the Colorado School of Mines [18]. In both reviews, there is information about Chenobi simulants. This is significant due to two reasons: (i) it is recommended for geotechnical use and (ii) the AGK2010 simulant (developed by CBK PAN and AGH) has very similar properties [19].

In the context of sampling tools and excavator development, there has been significant progress in the last decade. An extensive review of the currently developed sampling and excavation technologies was conducted by Zhang et al. (2023) [20]. Just et al. (2020) [21] provided a parametric review of various types of laboratory models of mining equipment and excavation methods. To classify the excavation processes more effectively, a first differentiation is made between discrete and continuous excavators. Discrete excavators are characterized by the need to break contact with the soil between cuts to clear the cutting surface or to dump the excavated material. Examples of discrete excavators include front-end loaders, dozers, or backhoes, which can be further subdivided into scrapers and scoopers [22]. To date, several sampling devices and excavators have been developed by CBK PAN: the Chomik device for the Russian Phobos Grunt mission [23], the Rotary Hammering Device (RHD), also called Packmoon [24], and recently, the Rotary Clamshell Excavator (RCE), developed in the frame of the ESA-funded DIGGER activity [25]. The last one was used as a carrier of the RF sensor presented in this paper.

The measurement of the mass of the regolith acquired by a sampling tool or excavator is a key issue discussed in this paper. Such measurements traditionally rely on force measurement using strain-gauge-based or piezoelectric sensors, which operate through force comparison methods (before and after sampling). However, several limitations restrict their applicability in extraterrestrial environments. These limitations include their sensitivity to environmental conditions and the challenge of achieving stringent uncertainty requirements under space mission constraints.

One major challenge arises from the reduced gravity on celestial bodies such as the Moon or Mars, which significantly lowers the measurable force exerted by the regolith mass on the sensor. This issue becomes even more critical for missions targeting small celestial bodies, such as OSIRIS-REx or Martian Moon Exploration [26,27,28]. Achieving the required measurement accuracy—at least 25 g for regolith sampling devices (RSDs)—is difficult under these conditions. Furthermore, the accuracy of force sensors is influenced by factors like nonlinearity, hysteresis, and inter-channel coupling [29]. These factors typically introduce uncertainty levels starting at 0.01% of the full measurement range and can reach as high as 3%, depending on the application and design. Although sufficient for many terrestrial applications, this level of uncertainty is inadequate for space missions with more demanding requirements.

Moreover, sampling devices are frequently mounted as end-effectors on manipulator arms or similar deployment systems. The inherent mass and volume constraints of space missions further restrict the incorporation of hold-down and release mechanisms (HDRMs), which are traditionally used to reduce launch loads. As a result, the system must fulfill two conflicting demands: it must be stiff enough to withstand substantial launch loads—up to 65 GRMS (root mean square acceleration)—while being compliant enough to detect small variations in mass. As an example, during the development of the RCE, it was determined that achieving accuracy at the level of 0.0001% of the full scale was essential, particularly given the need for the sensor to endure launch loads without the support of an HDRM. Current sensors based on strain gauge technology are incapable of reaching this extraordinarily low error margin.

Electromagnetic (EM) waves in the radiofrequency (RF) range are not dependent on the stiffness of the structure required to survive launch loads and therefore this technique was identified as a promising one to solve the problem described above. EM signals are widely used for many different applications, such as wireless communications, radar systems, remote sensing using synthetic aperture radar (SAR) mounted on aircrafts or satellites, and wireless contactless measurements of the properties of various types of materials—i.e., RF sensors. The idea of RF sensors is based on the interaction of the EM wave with objects placed in the path of the propagating wave or in close proximity to an element conducting the EM wave. Each RF sensor consists of two parts: a sensing element used to generate and receive the EM wave within the measured object or material (typically an antenna, resonator or transmission line) and an electronic module that generates the RF signal and measures the parameters of the signal received from the sensing element. RF sensors are low cost, compact, energy efficient and easy to integrate solutions that are used to measure and monitor the parameters of materials/objects (physical, chemical, or biological) in a non-invasive and non-destructive manner. Such sensors are used in many different sectors [30,31,32,33,34]: chemical engineering, civil engineering, the transport and food industries, healthcare, environmental monitoring, defense, security, and space applications. In our case, an RF sensor was developed and optimized for use in an existing lunar regolith sampling device, the RCE, to detect the mass of the acquired regolith, which is the main contribution described in this paper.

In the following sections of this paper, two main topics are described: (i) the proof of concept for the RF sensor operation, which is dedicated to space excavators and regolith sampling devices such as the RCE, and (ii) the results of a test campaign where the accuracy of the sensor was evaluated.

## 2. Materials and Methods

### 2.1. Rotary Clamshell Excavator Description

**Figure 1:**
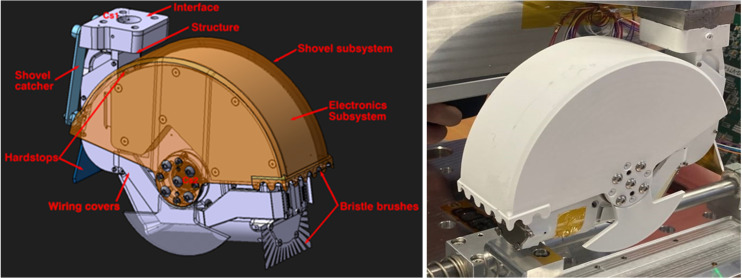
Main subsystems and components of the RCE in the isometric view. CAD model (**left**) and prototype (**right**).

The RCE is a single degree of freedom (DoF) electromechanical device operating with a low and constant velocity, with the main function being to sample lunar regolith [25]. The device is able to collect regolith from the lunar surface up to 30 cm in depth in a sequence of multiple operations. During testing, such a depth was reached on average in 24 single acquisition operations with a 90° attack angle. The RCE’s average operation (sample acquisition) time is 47 s. The maximum regolith sample weight is 0.31 kg and may vary depending on the surface topology and regolith relative density. During multiple operations up to 30 cm in depth, 6.5 kg of regolith was collected. The device is able to operate in highly cohesive and abrasive lunar regolith while limiting the vertical reaction forces to 18 N (in nominal relative density of regolith). The RCE is able to operate with an angle of attack in the range of 50° to 90° (vertical position) in lunar environmental conditions (temperature range: −100 °C to +105 °C and a high vacuum). The mass of the device is 2.1 kg and its power consumption is 8 W on average. The general dimensions of the system are 144 × 172 × 60 mm. The RCE has a TRL 6 level according to ECSS norms. A CAD model and an engineering protype of the RCE are presented in Figure 1.

**Figure 2:**
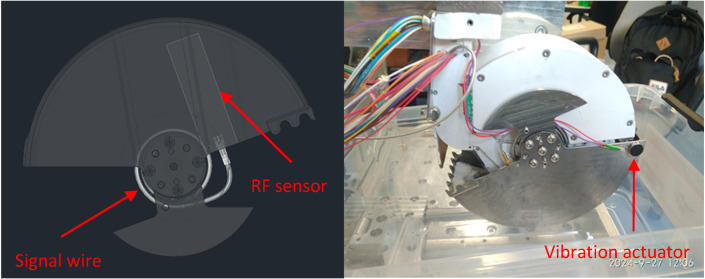
Placement of the RF sensor in the RCE shovel (**left**) and the vibration actuator (**right**).

The shovel was designed to enclose the entire mechanism to allow for a maximum possible footprint and thus maximize the trenching capability of the RCE. It has a cylindrical shape with a 166 mm diameter, enabling a depth of approximately 40 mm per single cut. The shovel is made of titanium alloy to reduce its mass, and counterweights made of tungsten are attached to centralize its center of gravity (CoG) close to the axis of rotation. The shovel’s teeth were designed based on mining principles—specifically, the teeth have slightly greater thickness to reduce the need for regolith compression inside the shovel. The shovel can be equipped with an RF sensor to estimate the mass of the collected regolith and a vibration system for leveling the regolith after the acquisition operation (Figure 2).

### 2.2. Concepts of the RF Sensor

Detection of the regolith inside the shovel using the RF sensor is based on the fact that regolith is a dielectric material, and its electric permittivity differs from that of vacuum and air. When the electromagnetic (EM) wave propagates in the regolith, its wavelength is shorter than the wavelength of the same wave propagating in a vacuum. This difference is leveraged to determine the thickness of the regolith layer inside the shovel and estimate its amount. Two preliminary solutions for the RF sensing element were considered:

- A patch antenna placed on top of the shovel, designed to transmit the EM wave inside the shovel and receive the reflected wave.
- A microstrip line with an open (or short) end, placed on the inner wall of the shovel and immersed in the regolith.

In both cases, the sensing element is connected to an electronic module that generates a radio frequency signal, sends it to the sensor, and receives the reflected wave from the sensor.

**Figure 3:**
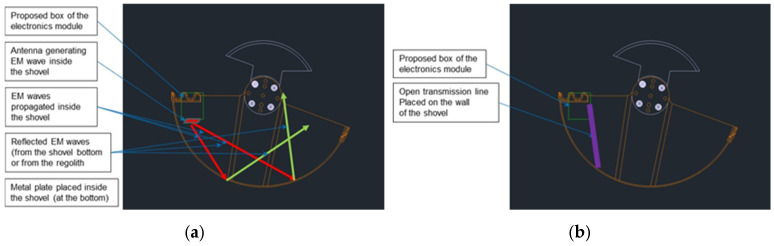
Possible detection methods for the regolith inside the shovel using radiofrequency waves: (**a**) sensor with an antenna; and (**b**) sensor with a transmission line (reflection of the wave in the line from the open or short end).

Both proposed RF sensor solutions are presented in Figure 3.

When the antenna is used as the RF sensor, the EM wave radiated by the antenna propagates inside the shovel toward its bottom, and after reflection, a portion of the reflected wave is received by the antenna. The phase of the reflected wave depends on the thickness of the regolith inside the shovel. Detection of the phase change compared to the empty shovel, performed by the electronic module, can be utilized to estimate the amount of regolith.

**Figure 4:**
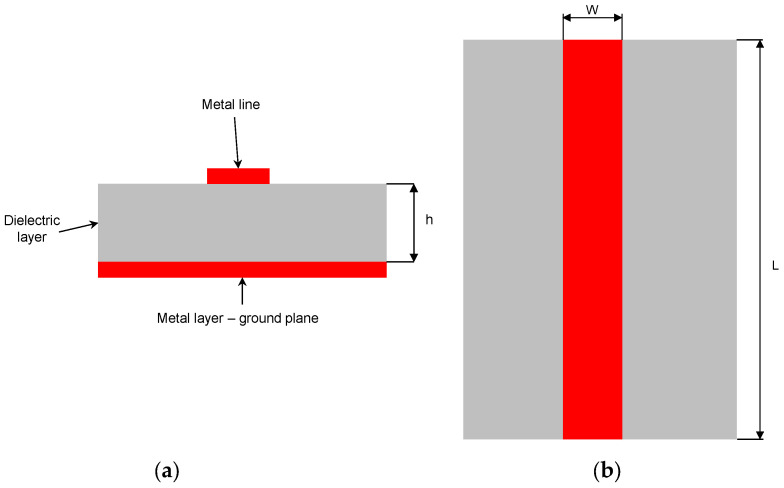
Construction of the microstrip line: (**a**) cross-sectional view; and (**b**) top view.

The microstrip line is a planar structure consisting of three layers (Figure 4):

- A metal ground plane at the bottom.
- A dielectric layer in the middle.
- A metal strip at the top.

The impedance of the microstrip line depends on the electric permittivity of the used dielectric material, its thickness (h) and the width of the metal strip (W). For a given type of dielectric laminate, the width of the strip is selected to obtain a given value of the impedance of the line, ensuring impedance matching with the circuits to which it is connected. Typically, this is a value of 50 ohms.

The wavelength of the EM wave propagating in the line depends on the effective permittivity of the line, which is a function of the parameters of the dielectric laminate, the dimensions of the line and the frequency of the wave. In general, the higher the permittivity of the laminate, the shorter the wavelength. The phase shift of an EM wave propagating in the line between the end and the beginning of the line depends on the length of the line L relative to the wavelength. Since the wavelength is inversely proportional to the wave frequency, a larger phase shift is observed at higher frequencies for the same line length.

**Figure 5:**
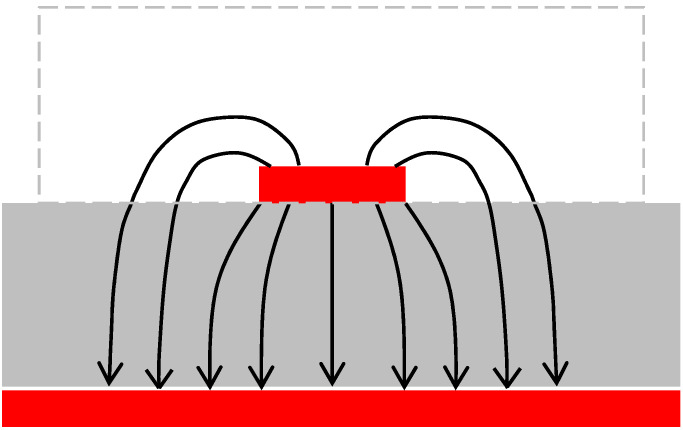
Electric field distribution for the EM wave propagating in the microstrip line. The dashed rectangle indicates the area where placing a dielectric material with electric permittivity different from air/vacuum will significantly affect the parameters of the EM wave propagating in the line.

When the microstrip line is used as the RF sensor, the EM wave, generated by an electronic module, propagates along the line, and after reflection at the open (or short) end (reflected wave), it returns back to the input port, causing a standing wave in the line. For open-type lines, such as the microstrip line, the EM field distribution around the line (illustratively presented in Figure 5) depends on the parameters of objects in the immediate vicinity of it (within the area of the dashed rectangle in Figure 5).

**Figure 6:**
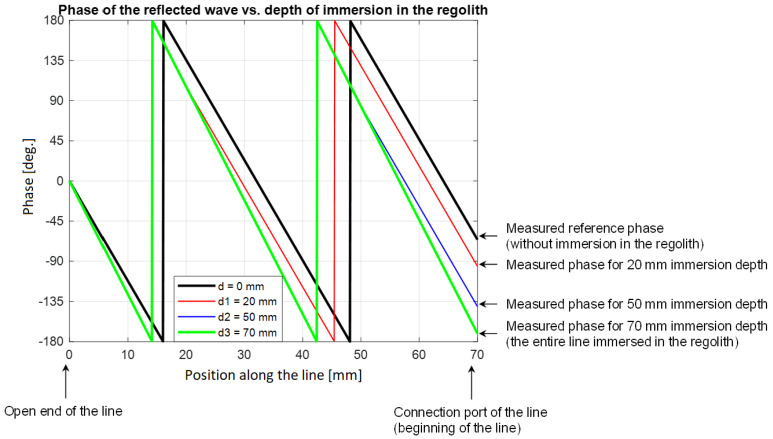
The phase of the EM wave reflected from the open end of the line and propagated back to the beginning of the line.

Immersion of the line in the regolith (in general, in a dielectric material with electric permittivity higher than that of air/vacuum) changes the parameters of the waves propagating in the line, which translates into a phase change of the reflected wave. The phase change is proportional to the immersion depth of the line into the regolith and the frequency of the EM wave. Figure 6 presents the phase of the reflected wave propagating in the line in dependence on the immersion depth of the line in the regolith (for a frequency of 5 GHz and a line length of 70 mm). The arrows on the right show the values of the phase measured at the beginning of the line (port connecting the line to the electronic module). The phase change also depends on the permittivity of the material in which the line is immersed.

Similar to the solution with the antenna, detection of the phase change by the electronic module allows estimation of the amount of the regolith inside the shovel. The phase change of the reflected wave is proportional to the depth of immersion of the line into the regolith, so the microstrip line should be placed on the inner side of the shovel from the upper edge toward the bottom.

The preliminary planned location of the sensor’s electronic module is marked in Figure 3. If the antenna is used as the RF sensor, it will be permanently attached to the electronic module. However, in the case of the microstrip line, which will be mounted on the inner side of the shovel wall and will move with it during regolith acquisition, it is necessary to ensure a connection between the line and the electronic module using a coaxial cable. The length of this cable and its routing must maintain the connection for all the positions of the shovel while minimizing the impact of position changes on the measurement results.

**Figure 7:**
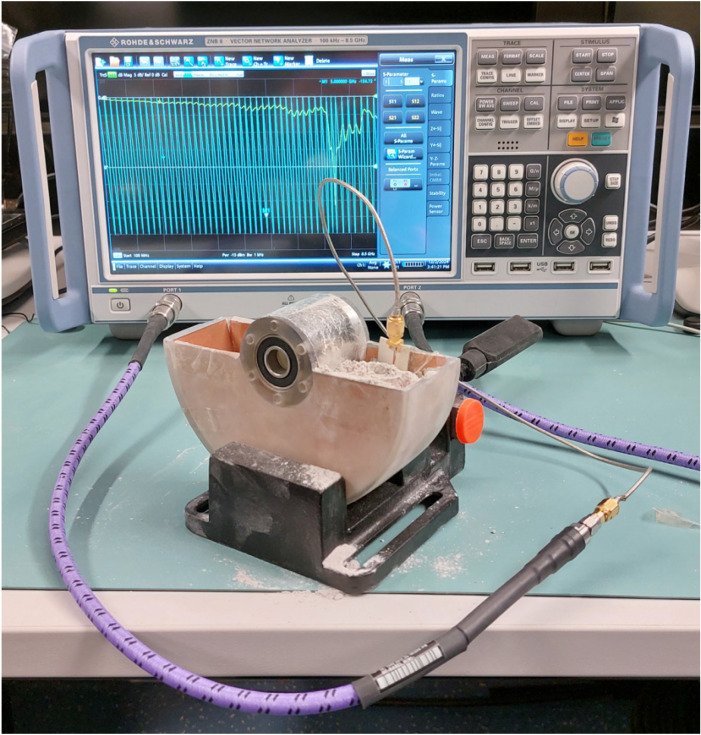
Measurement setup for the preliminary test of the microstrip line as an RF sensor.

Preliminary measurement tests for both aforementioned solutions of the RF sensor were performed using the model of the shovel and the vector network analyzer ZNB 8 (Rohde Schwarz, Munich, Germany) as an RF signal generator and detector for the reflected wave phase (S11 parameter of the scattering matrix). The measurement setup is presented in Figure 7. Due to the shape of the shovel (presented in Figure 3), which caused a small amplitude of the reflected EM wave received by the antenna used as the sensor, the solution with the microstrip line as the RF sensor was chosen.

To determine the optimal location for the microstrip line inside the shovel, tests involving scooping regolith into the shovel were carried out. After each scooping action, the distribution of the regolith inside the shovel was observed. Tests showed that the regolith tends to break down and accumulate in the part of the shovel immersed during acquisition. Therefore, the microstrip line should be placed on the inner wall of the shovel in this part (Figure 3b).

Regardless of which type of RF sensor is used to estimate the amount of acquired regolith, the phase of the reflected EM wave depends on the electric permittivity of the regolith. To use the RF sensor to detect the amount of regolith in the shovel, it is necessary to perform calibration measurements using an appropriate regolith simulant. During the tests and measurements presented in the following sections, the AGK2010 simulant was used.

The model of the shovel was used in the preliminary tests. The 50 Ω microstrip line, with a length of 70 mm, was made using microwave laminate RO 4003C with a thickness of 0.7 mm and electric permittivity of 3.5. The RF sensor has dimensions of 70 mm × 17 mm, with the microstrip line length being 70 mm and the width of the strip on the top of laminate being approximately 1.6 mm. The line was glued to the inner wall of the shovel, in the part immersed in the regolith during scooping. The length of the microstrip line was chosen such that its open end was located near the bottom of the shovel, while the second end, connected to the vector network analyses, was positioned above the expected maximum height of the regolith in the shovel.

**Figure 8:**
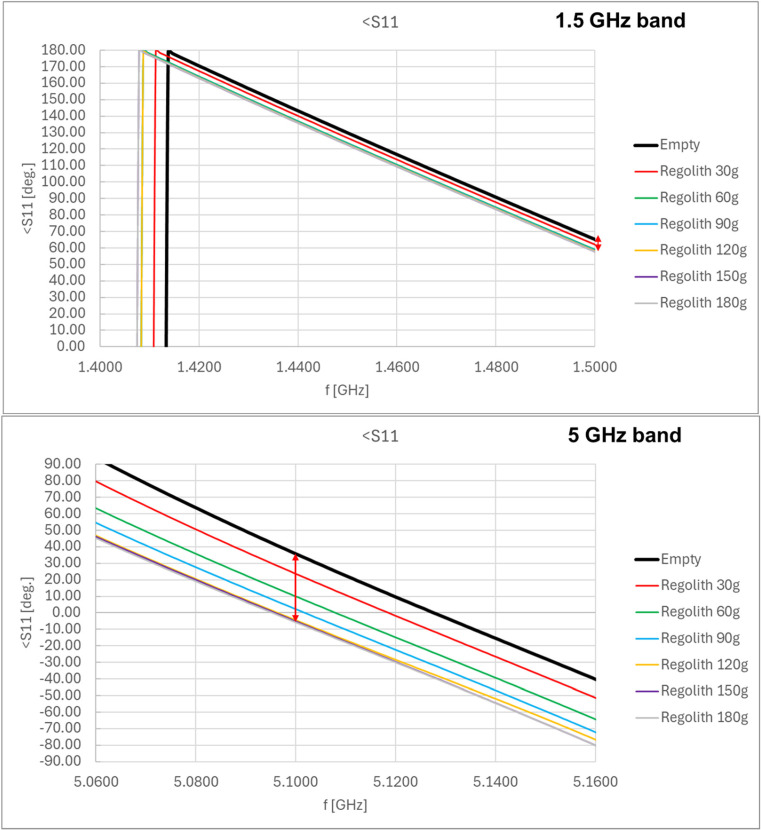
Example results of the preliminary measurements for the microstrip line as the RF sensor: phase of the reflected wave (coefficient S11 of the scattering matrix) as a function of the amount of regolith inside the shovel for the 1.5 GHz and 5 GHz RF signal bands (red arrows show the range of the phase change in each band, depending of the regolith mass).

The inside of the shovel model was lined with a copper sheet to simulate the metal walls of the shovel. Example results from the preliminary tests, showing the change in the phase of the reflected wave at the beginning of the line (S11 coefficient of the scattering matrix measured by vector network analyzer), are presented in Figure 8 for different amounts of regolith inside the shovel and for two frequencies, 1.5 GHz and 5 GHz. The results showed that changes in the phase of the S11 coefficient (reflected wave), caused by the presence of regolith inside the shovel, were observed.

Larger changes were observed at higher frequencies of the signal fed to the microstrip line. However, the signal frequency should not be too high to ensure the ease of implementation of the electronic module connected to the RF sensor. For the 5 GHz band, the complete immersion of the microstrip line into the regolith resulted in phase changes in the S11 coefficient within a range of 40–50°, compared to the value for the empty shovel. For the 1.5 GHz band, the changes were limited to several degrees. Based on these results, the 5 GHz band was selected for use in the proposed sensor.

### 2.3. Proof of RF Sensor Concept Operation

**Figure 9:**
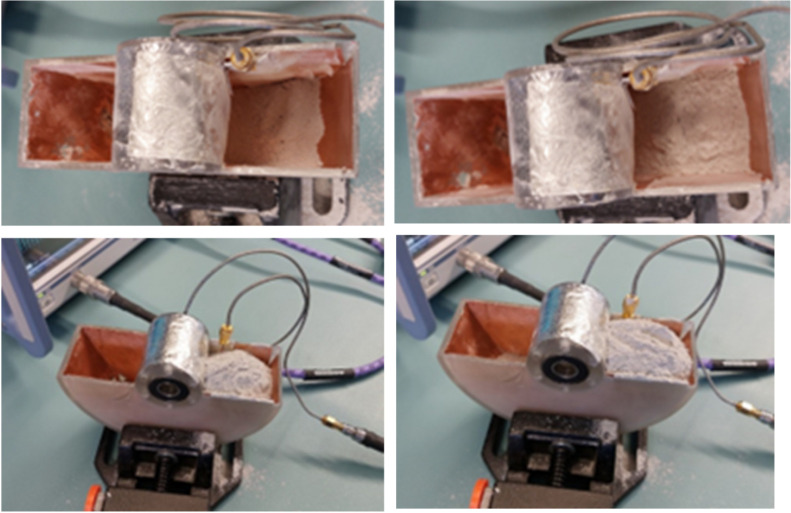
Measurements with different amounts of regolith inside the shovel.

To estimate the accuracy of determining the amount of regolith in the shovel using the microstrip line inside the shovel as an RF sensor and the proposed detection method of measuring the phase changes in the reflected wave, several series of measurements were performed. In each series, measurements were made for an empty shovel and a shovel with different amounts of regolith poured into the shovel: ranging from 30 g to 240 g, in 30 g increments. Figure 9 presents images of the shovel with varying amounts of regolith poured into it.

**Figure 10:**
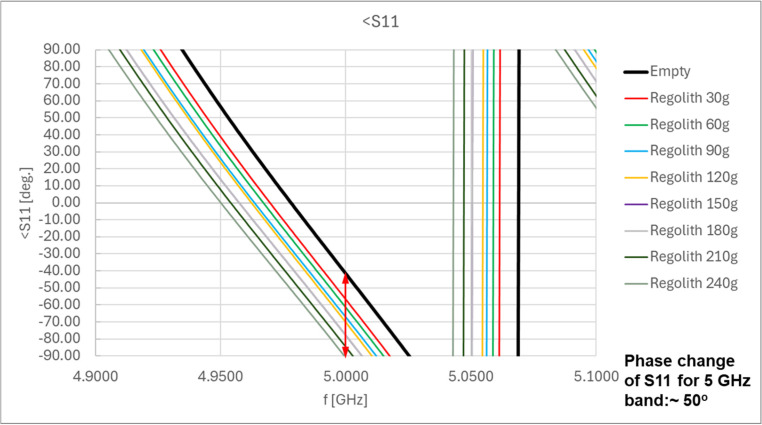
Results of the measurements of the reflection coefficient S11 phase for the EM wave propagating in the microstrip line for a single measurement series—different amounts of regolith inside the shovel (5 GHz RF signal band)—the red arrow shows the range of the phase change depending of the regolith mass.

An example of the results obtained in a single measurement series is shown in Figure 10 for a 5 GHz frequency of the RF signal. Depending on the amount of regolith in the shovel, changes in the phase of the S11 coefficient of the scattering matrix (reflected wave) in the range of approximately 50 degrees were observed.

Measurements were conducted for two types of series:

- Series S—where the regolith was poured into the shovel without spreading or smoothing it inside the shovel.
- Series R—where the regolith was poured into the shovel and then smoothed by shaking or using a spoon to ensure even distribution inside the shovel.

**Figure 11:**
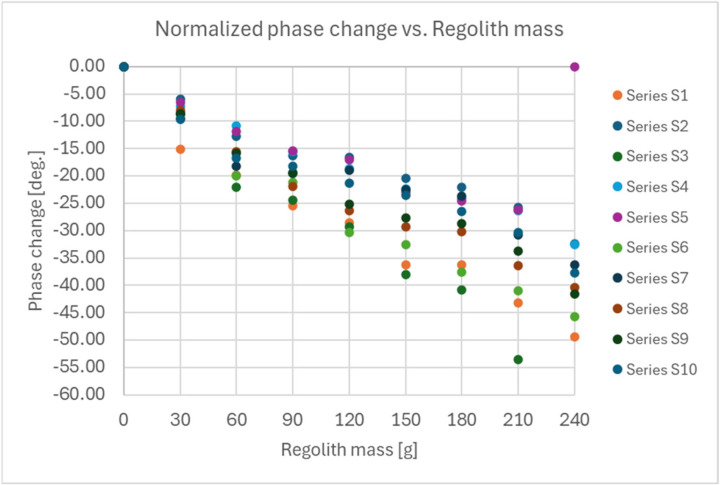
Results of the measurements of the reflection coefficient S11 phase for Series S—regolith in the shovel without smoothing (for 5 GHz RF signal frequency).

**Figure 12:**
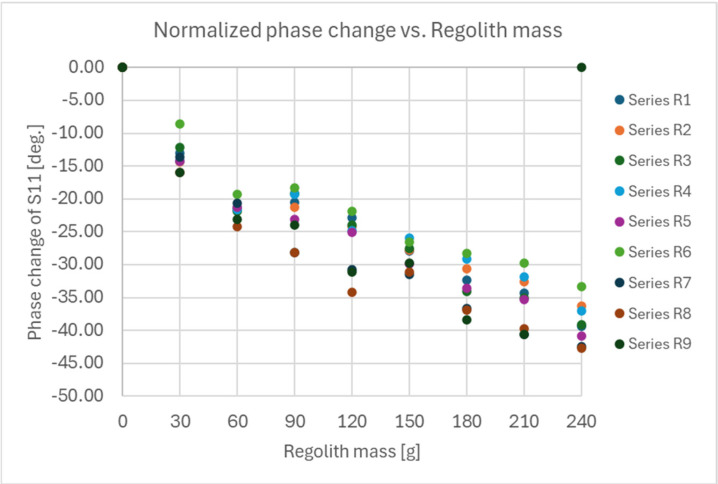
Results of the measurements of the reflection coefficient S11 phase for Series R—regolith smoothed in the shovel (for 5 GHz RF signal frequency).

The results for both types of series are presented in Figure 11 for measurements without smoothing and in Figure 12 for measurements with smoothing of the regolith inside the shovel.

Based on these results, a model of the relationship between the measured phase and the mass of the regolith inside the shovel was derived using polynomial fitting.

The statistics of the obtained results of the measurements for both types of series are presented in tables: Table 1, which considers the mean values and standard deviations of the measured phase for each amount of regolith inside the shovel, and Table 2, which considers the mean values and standard deviations of the phase difference relative to the result measured for the empty shovel in a given series.

The following conclusions were drawn from the presented results:

- Determination of the amount of the regolith inside the shovel should be performed in two steps: a measurement for the empty shovel before scooping and a measurement after scooping the regolith into the shovel, considering the difference in the measured phase of S11 relative to the value for the empty shovel.
- Smoothing the regolith inside the shovel after scooping can significantly increase the accuracy of estimating the amount of the regolith in the shovel (two to three times smaller standard deviation of the measured phase difference of the S11 coefficient compared to the empty shovel).
- The accuracy of the mass estimation was evaluated using a linear approximation function, validated against a separate dataset that was not included in the calibration process. The analysis revealed an average absolute error of 8.0 g in the estimated regolith mass. Correspondingly, the average relative error was calculated to be 10.2%.

## 3. Results

### 3.1. Measurement Setup

The RCE engineering model (Figure 1), with a sample sensor attached to the shovel interior, was used in the conducted experiments. The shovel was also equipped with a vibration motor to level the regolith after sampling operations. Both the sample sensor and the vibration motor have a lower TRL level.

**Figure 13:**
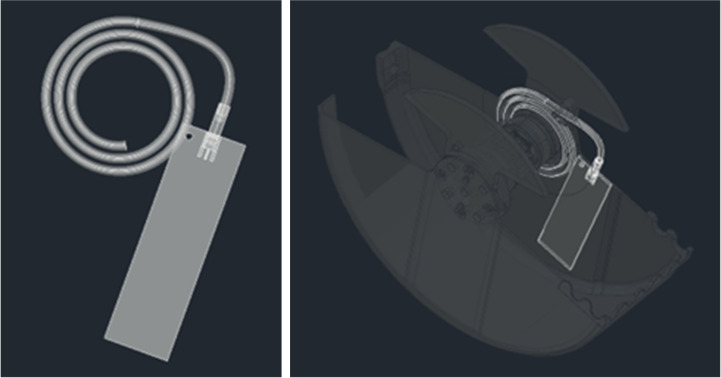
Model the RF sensor and its location inside the shovel.

The 50 Ω microstrip line, used as the RF sensor, was made using Taconic RF35 laminate (Taconic, Petersburgh, NY, USA) with an electric permittivity of 3.5. The sensor was glued to the inner wall of the shovel, and a coaxial cable was connected to the line. The cable was wound around the axis of the shovel, allowing continuous connection to the electronic module in all the positions of the shovel. A model of the shovel with the proposed location of the microstrip line and coaxial cable is presented in Figure 13.

**Figure 14:**
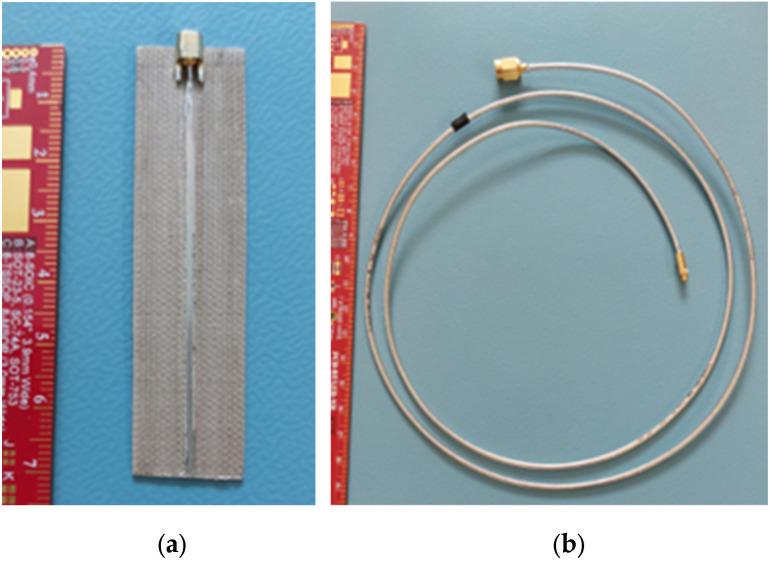
RF sensor elements: (**a**) microstrip line as the RF sensor; and (**b**) coaxial cable to connect the RF sensor to the electronic module.

The dimensions of the laminate with the microstrip line were 69 mm × 19 mm, with a thickness of less than 1 mm. The coaxial cable RG-405, approximately 1 m in length, was used to connect the microstrip line to the electronic module. The connection between the line and the cable was established using MMCX connectors (jack for the line and plug for the cable). For the second end of the cable, an SMA plug was used. The microstrip line and coaxial cable are presented in Figure 14.

Measurement validation of the proposed method for estimating the regolith quantity inside the shovel was conducted during a scooping campaign performed at the Space Research Centre PAS. The experiment was carried out in a standard laboratory environment where the temperature and humidity were neither explicitly controlled nor monitored. Based on typical laboratory conditions, the ambient temperature is assumed to have ranged between 20 and 25 °C, with relative humidity levels of 30 to 60%. These assumptions align with standard indoor environmental parameters and provide context for interpreting the experimental results.

**Figure 15:**
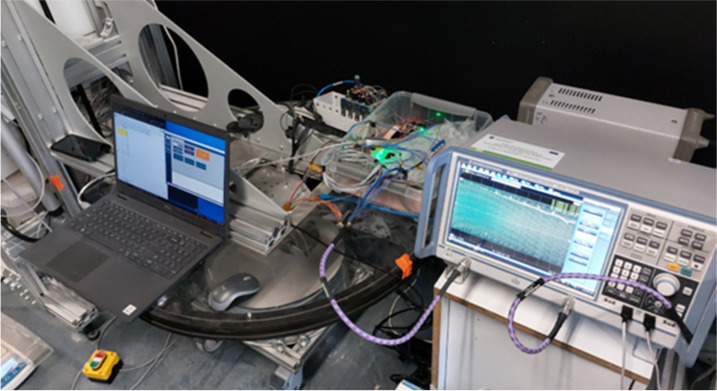
Measurement setup for validation of the regolith quantity detection using the microstrip line as the RF sensor.

The measurement setup is presented in Figure 15. The vector network analyzer RS ZNB 8 was used to determine the phase of the reflected wave propagated in the microstrip line (reflection coefficient S11) glued to the inner wall of the shovel. Each measurement consisted of two parts, allowing for determination of the phase change caused by the presence of regolith inside the shovel:

- Measurement of the S11 phase for empty shovel—used for determining the reference phase.
- Measurement of the S11 phase for the shovel with regolith inside after its acquisition.

Based on the results of both measurements, the phase change was determined for each regolith acquisition. After each acquisition and measurement, the regolith from the shovel was weighted.

During the campaign, a total of 35 regolith acquisitions were performed. For each acquisition, two measurements were conducted:

- Regolith without shaking (before shaking).
- Regolith after shaking.

**Figure 16:**
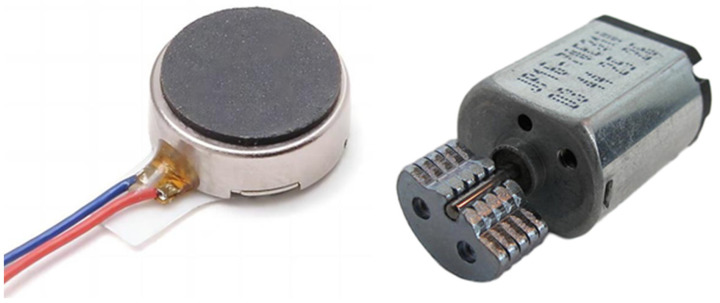
Photographs of the vibration motors evaluated in this study: JYC1234 (**left**) and VJP16-70E310 (**right**).

As the vibration effector, two miniature DC motors were evaluated for suitability (Figure 16):

- JYC1234 (JIE YI Electronics Limited, Shenzen, China): A 3 VDC coin motor with a rotational speed of 12,000 rpm.
- VJP16-70E310 (Vybronics, Hong Kong, China): A 7.2 VDC vibration motor with a rotational speed of 8250 rpm.

**Figure 17:**
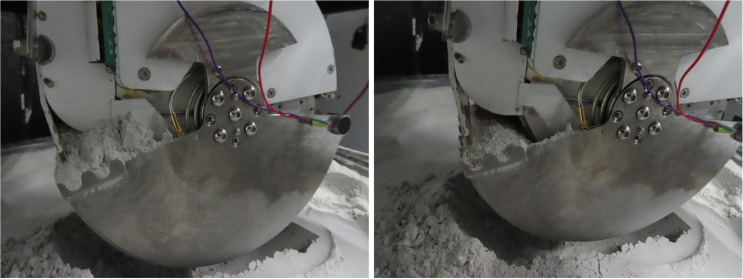
Regolith sample inside the shovel before shaking (**left**) and after 10 s of shaking (**right**) using the vibration motor.

For the experiment, the VJP16-70E310 motor was selected due to its performance characteristics. Following the acquisition of each regolith sample, and after the initial phase shift was recorded, the VJP16-70E310 motor was activated for a duration of 10 s. The regolith displacement induced by the vibration motor is shown in Figure 17. Subsequently, the final phase shift was measured to assess the impact of the vibration.

### 3.2. Measurement Results

**Figure 18:**
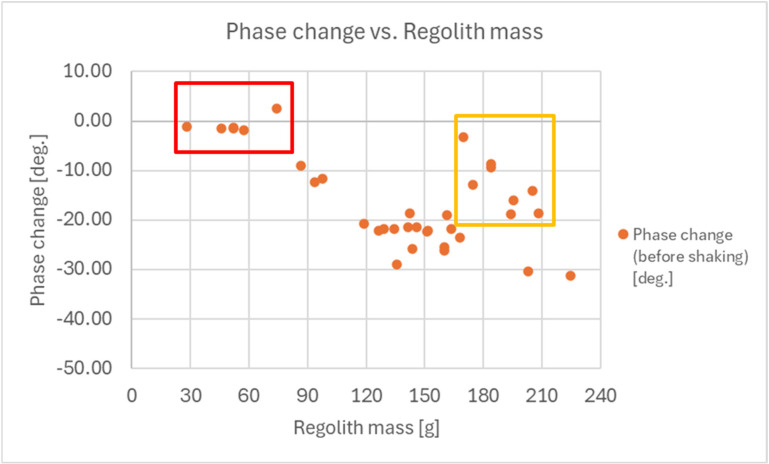
Results of the measurements of the reflection coefficient S11 phase for the performed acquisitions of the regolith into the shovel (for a 5 GHz RF signal frequency) without shaking.

The results for all the acquisitions of the regolith without shaking (before shaking) are presented in Figure 18, which shows the dependence of the measured phase change on the weight of the regolith scooped into the shovel. Two deviations from the expected proportional relationship between the measured phase change and regolith mass can be seen in the figure:

- No phase changes for a small amount of the regolith in the shovel, less than approximately 80 g—this weight range is marked using red color. Because of the few-millimeter-wide gap between the end of the RF sensor and the bottom of the shovel, a small quantity of regolith inside the shovel does not cause a significant phase change, resulting in a sensor dead zone.
- Slight phase changes for large amount of the regolith inside the shovel, exceeding approximately 170 g. This weight range is marked in brown. In this case, the regolith accumulates in the part of the shovel that was immersed during acquisition, and the RF sensor is not adequately immersed in the regolith. This causes the measured phase change to deviate from the actual amount of regolith inside the shovel.

Shaking of the regolith inside the shovel after acquisition reduces the influence of the abovementioned problems on the determination of the regolith amount and improves the accuracy of estimating the mass of the acquired regolith.

**Figure 19:**
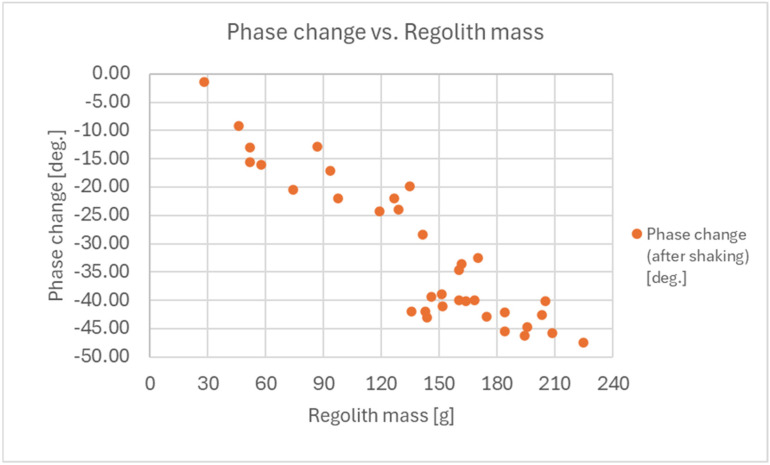
Results of the measurements of the reflection coefficient S11 phase for the performed acquisitions of the regolith into the shovel (for a 5 GHz RF signal frequency) after shaking.

The results for shaking the regolith after acquisition are presented in Figure 19.

Using the collected data points, which demonstrate a correlation between the actual mass *mass_r* of the sampled regolith and the measured phase shift, a linear regression estimating the regolith sample mass *mass_e* was derived using the least squares method.


**Figure 20:**
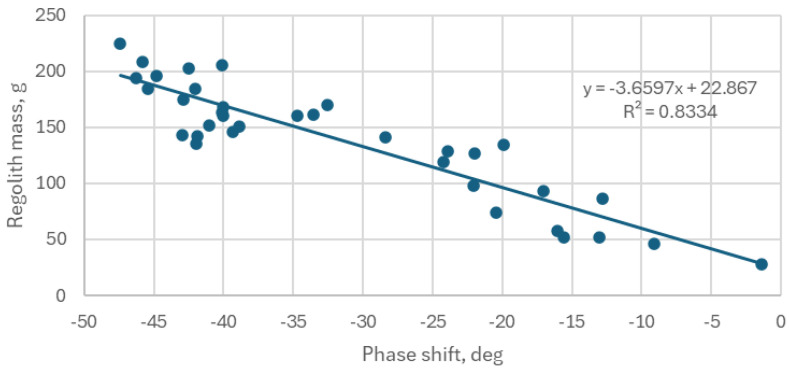
Acquired data points plotted along with the determined estimation function.

(1) mass_e = a × phase_shift +  b,

The coefficients of the calibration function are *a* = −3.6597 and *b* = +22.87. The plot of the measurement data and the obtained linear fit is shown in Figure 20.

To validate the reliability of the regression model used for the mass estimation, a statistical analysis was conducted on the obtained regression coefficients. A hypothesis test (H_0_) was performed to evaluate the significance of these coefficients, and the *p*-values were calculated for each. The results indicated that both *p*-values were well below the 5% threshold, confirming the statistical significance of the regression model. Furthermore, the standard error and 95% confidence intervals for the regolith mass estimation function were calculated. These statistical metrics are summarized in Table 3 and Table 4.

To quantitatively assess the achieved accuracy of the mass estimation, the percentage relative difference (*PRD*) method was employed. The *PRD* was calculated using the following formula:

(2) $$PDR=\frac{1}{n}\sum \left|\frac{m_{r}-m_{e}}{m_{r}}\right|\cdot 100\%=15.0\%,$$

where *m_r_*—actual mass and *m_e_*—estimated mass.

Moreover, the standard deviation of the estimation relative error was also determined using the following formula:

(3) $$STD_{DEV}=std\left(\left|\frac{m_{r}-m_{e}}{m_{r}}\right|\cdot 100\%\right)=12.6\%,$$

The average *PRD* across all the samples was found to be 15.0%, indicating the relative accuracy of the mass estimation using the described sensor.

## 4. Discussion

The discussion provided here is limited to a comparison of the results obtained with and without vibration applied to shovel. Specifically, there is no comparison to other mass measurement systems (e.g., based on a strain gauge) since there is no clear information on the accuracy of such sensors applied to ongoing or past space missions.

The measurement without applying vibrations to the regolith demonstrated notable precision, achieving a percentage relative difference (PDR) of 6.7% within a narrowed mass range of 80–165 g. However, the analysis identified two primary limitations that constrain the measurement range.

The lower limit is dictated by the distance between the microstrip line and the edge of the shovel wall. When small amounts of regolith are sampled, the material remains positioned too far from the RF sensor, resulting in a signal that is insufficiently strong for accurate detection. A potential solution to this limitation would involve redesigning the microstrip line layout to allow its placement closer to the edges of the shovel wall, thereby enhancing the sensitivity to smaller regolith masses.

The upper limit of the measurement range is affected by the bulk formation of regolith at the entrance of the shovel when larger samples are taken. In these cases, not all of the regolith enters the shovel, and the portion remaining outside does not significantly influence the sensor’s readings. To extend the upper limit of the measurement range, it would be more effective to focus on mechanisms that facilitate the complete fall-in of regolith into the shovel, rather than modifying the microstrip line layout. The initial test using a small vibration motor showed promising results in extending the upper limit of the measurement range.

Inducing vibration after regolith acquisition causes the material to settle deeper within the shovel. The analysis reveals that all the samples of similar mass collected with vibration produce phase shift readings that are closely clustered. Consequently, the range limits for the mass estimation were extended, as the measurement points in the phase shift–mass space aligned more closely with the linear approximation line.

While the resulting accuracy, measured as the percentage relative difference (PDR), decreased to 15.0%, the application of vibration enables the determination of the captured mass across the entire considered acquisition range. This trade-off between reduced accuracy and an expanded operational range highlights the potential benefits of vibration-assisted mass estimation for broader applicability.

## 5. Conclusions

In this paper, the concept of an RF sensor dedicated to measuring the mass of the regolith acquired by a sampling device or excavator designed for a planetary environment was presented. The microstrip line with an open end was used as the sensing element. Measurements of the phase of the reflection coefficient (S11 of scattering matrix) for the line immersed in the regolith were used to estimate the mass of the regolith inside the excavator shovel. For the evaluation of the sensor performance, the Rotary Clamshell Excavator (RCE) was used. The device has a TRL 6 level, and it passed the environmental test campaign, which indicates that the device is well suited for future lunar missions. The following conclusions were reached during sensor development:

- It is possible to measure the acquired mass of regolith using RF signals with reasonable accuracy of around 15%, which is good enough for rough regolith mass estimation in sampling devices or excavators.
- The regression model underwent statistical testing, and the obtained *p*-values for the regression coefficients confirmed their statistical significance.
- The RF sensor can be installed on a thin cross-section shovel without impacting the digging operation and performance.
- The vibration system improves the sensor accuracy; however, its mounting on the shovel implies a need for additional cable routing and an increase in the shovel thickness.
- The use of vibrations extends the measurable mass range, enabling the estimation of both smaller and larger sample masses beyond the limits of static measurement methods.
- The proposed system is lightweight and scalable, making it suitable for integration into various planetary excavation systems where the mass constraints are critical.
- The real lunar regolith might behave differently than the lunar analogue since analogues are developed to replicate only some features of real regolith; however, as lunar regolith is a dielectric material, the key principles of the RF sensor operation, like the signal phase shift, should be applicable to real cases.

Future development is needed to increase the TRL level of the RF sensor. Particularly, it is planned to realize two types of activities:

- Tests in reduced gravity conditions to validate both the RCE performance as well as the RF sensor accuracy with respect to this important difference between terrestrial and lunar environments.
- Development of dedicated RF sensor front-end electronics suitable for the RCE electronics box compartment shown on Figure 1.

## Figures and Tables

**Table 1:** Statistics of the results of the measured phase of S11 for both types of series.

Regolith Mass [g]	Mean Value (Without Smoothing)	Standard Deviation (Without Smoothing)	Mean Value (With Smoothing)	Standard Deviation (With Smoothing)
0	−46.4	3.8	−54.6	2.1
30	−55.3	4.2	−68.1	2.3
60	−62.8	6.3	−76.2	1.8
90	−66.2	6.0	−76.9	4.3
120	−69.7	7.6	−81.0	4.6
150	−74.0	8.2	−83.2	3.0
180	−75.9	8.6	−87.8	4.0
210	−81.2	11.1	−90.1	4.4
240	−88.6	11.3	−93.2	3.2

**Table 2:** Statistics of the results of the measured phase difference relative to the measurement for the empty shovel for both types of series.

Regolith Mass [g]	Mean Value (Without Smoothing)	Standard Deviation (Without Smoothing)	Mean Value (With Smoothing)	Standard Deviation (With Smoothing)
0	0	0	0	0
30	−8.8	2.5	13.6	2.3
60	−16.4	3.8	21.7	1.4
90	−19.8	3.5	22.4	3.7
120	−23.2	5.3	26.5	4.3
150	−27.6	6.2	28.7	2.0
180	−29.5	6.6	33.3	3.5
210	−34.7	9.0	35.5	4.0
240	−42.0	9.5	38.9	3.2

**Table 3:** Statistics of the regression model.

Parameter	Value
Multiple R	0.913
R^2^	0.833
Standard error, g	21.375
Observations	35

**Table 4:** Statistics of the obtained coefficients.

	*b*	*a*
Coefficient value	22.867	−3.660
Standard error	9.731	0.285
T statistic	2.350	−12.849
*p*-value	0.025	2·10^−14^
Lower 95%	3.070	−4.239
Upper 95%	42.664	−3.080

## Data Availability

The data forming the basis of the presented study might be available from the authors upon reasonable request.
